# Boundary-Sensitive Hybrid Attention Network for Multi-Scale Crack Fine Segmentation

**DOI:** 10.3390/s26103200

**Published:** 2026-05-19

**Authors:** Yaotong Jiang, Tianmiao Wang, Congyu Shao, Xuanhe Chen, Jianhong Liang

**Affiliations:** 1 School of Mechanical Engineering and Automation, Beihang University, Beijing 100191, China; yaotong1995@buaa.edu.cn (Y.J.); itm@buaa.edu.cn (T.W.); shaocongyu@buaa.edu.cn (C.S.); 2 School of Physical Science And Technology, Beijing University of Posts and Telecommunications, Beijing 102206, China; 2010815252@bupt.cn

**Keywords:** bridge health monitoring, crack segmentation, Transformer encoder, multi-scale features, boundary-aware decoder

## Abstract

**Highlights:**

A novel Boundary-Sensitive Hybrid Attention Network (BSA-Net) is proposed for fine-grained, multi-scale crack segmentation in concrete bridge health monitoring. The model integrates a hierarchical Transformer encoder, a multi-scale context enhancement, and a boundary-aware dual-branch decoder to improve segmentation accuracy and boundary clarity.

**What are the main findings?**

- The BSA-Net model achieves competitive performance on existing crack detection models, demonstrating enhanced performance in complex environments with weak contrast and noisy backgrounds.
- The proposed model improves segmentation accuracy, particularly in fine-scale crack detection, by leveraging multi-scale feature extraction and boundary refinement mechanisms.

**What are the implications of the main findings?**

- BSA-Net offers a robust and scalable solution for automated infrastructure monitoring, providing accurate and reliable crack segmentation in real-world applications.
- The method can be adapted for use in other engineering fields that require high-precision segmentation of fine details in challenging imaging conditions.

**Abstract:**

Concrete crack segmentation in bridge health monitoring is crucial for ensuring the safety and longevity of infrastructure. However, this task is complicated by challenges such as weak contrast, background interference, and multi-scale crack structures, which hinder traditional methods’ accuracy. This study introduces a novel Boundary-Sensitive Hybrid Attention Network (BSA-Net) designed to address these issues by combining a hierarchical Transformer encoder (Hiera-A), a multi-scale context module (Light-ASPP), and a boundary-aware decoder (BAD). The hierarchical encoder effectively captures multi-scale features, while Light-ASPP enhances the network’s ability to aggregate contextual information with minimal computational cost, making it suitable for large-scale applications. The dual-branch decoder explicitly decouples the learning of semantic segmentation and boundary prediction, ensuring more accurate boundary detection and crack continuity. The extensive experiments on multiple benchmark datasets demonstrate that BSA-Net consistently outperforms existing crack detection models, particularly in complex, noisy environments. The model achieves competitive performance in terms of segmentation accuracy, boundary clarity, and recall rates, particularly for fine-scale and weak contrast cracks. The results indicate that BSA-Net not only enhances the performance of crack segmentation in real-world conditions but also provides a scalable and reliable solution for automated infrastructure monitoring and defect detection.

## 1. Introduction

As a crucial component of transportation infrastructure, the service safety of bridges directly impacts traffic efficiency and public safety. Surface cracks are one of the most common and visible manifestations of damage in concrete bridges under the combined effects of long-term load, environmental erosion, and temperature-humidity cycles. The formation and propagation of cracks not only reduce the structural durability but may also indicate potential risks such as material degradation, stress concentration, and local capacity decline. Therefore, research on high-precision identification and segmentation of concrete cracks in bridges is an important foundation for bridge condition assessment, maintenance decisions, and risk warning [1,2,3,4]. Research related to bridge crack segmentation and measurement has also shown that pixel-level results can provide more reliable data support for subsequent quantification of crack width, length, and other parameters [4,5].

Traditional crack inspection mainly relies on manual visual inspection or image processing methods such as thresholding and edge detection. While these methods are somewhat effective in controlled environments, they face significant limitations under real-world bridge conditions. Bridge surfaces are often subject to interference from factors such as shadows, stains, joints, rough textures, and uneven lighting. Cracks and backgrounds frequently exhibit strong similarities in grayscale and texture, leading to false positives and missed detections [2,6,7]. Particularly, fine cracks, low-contrast cracks, and discontinuous cracks are more challenging to extract reliably in complex backgrounds, and traditional methods struggle to balance both robustness and precision [6,7,8].

From an engineering perspective, crack identification tasks not only require the judgment of “whether there is a crack,” but also emphasize the complete representation of the crack area and boundary localization, which supports subsequent disease quantification and structural evaluation. Compared to classification and object detection methods, semantic segmentation directly outputs pixel-level crack masks, which better align with the elongated, bifurcated, and multi-scale morphological features of bridge cracks. This has become the primary direction in current crack visual detection research [8,9,10]. Meanwhile, classic segmentation frameworks such as FCN, U-Net, and DeepLabV3+, along with their improved models, provide an effective technical foundation for crack segmentation [11,12,13]; the development of Transformer models and high-resolution modeling methods further enhances global modeling and cross-scale representation capabilities in complex scenarios [10,14,15,16].

In summary, semantic segmentation research for concrete cracks in bridges has clear engineering value and academic significance: on one hand, it can enhance the accuracy and stability of crack identification in complex backgrounds; on the other hand, it can provide a higher quality data foundation for crack geometric quantification, damage evolution analysis, and intelligent maintenance decision-making. In response to the common issues in bridge scenarios such as “strong background interference, difficulty in detecting fine cracks, and blurred boundaries,” the design of models focused on fine-grained segmentation is both necessary and practically meaningful.

Crack visual detection methods have generally evolved from traditional image processing techniques to deep learning-driven approaches. Early methods largely relied on thresholding, edge detection, and morphological operations, which were sensitive to changes in imaging conditions and lacked stability under complex textures, shadows, and noise interference [6,9]. With the development of deep learning, crack recognition gradually shifted to data-driven methods and has been widely applied in scenarios such as roads, bridges, tunnels, and concrete structures [2,3,8,9,17].

In terms of task types, existing methods mainly include classification, object detection, and semantic segmentation. Classification methods are suitable for rapid screening but struggle to provide precise boundaries. Object detection methods can locate crack regions but have limited ability to express the shapes of elongated, curved, or discontinuous cracks. Semantic segmentation methods, by performing pixel-level classification, describe crack regions and are more suitable for detailed recognition and subsequent quantification analysis of bridge defects [8,9,10]. As a result, current research has gradually shifted towards crack detection frameworks centered around semantic segmentation.

In terms of network architectures, FCN, U-Net, and DeepLabV3+ are the most commonly used foundational frameworks in crack segmentation research [11,12,13]. Based on these, researchers have made numerous improvements focusing on multi-scale contextual modeling, attention mechanisms, and decoder designs. For example, research on crack segmentation in complex bridge scenarios reduces the feature similarity between cracks and background through feature separation and competition mechanisms [7]; some methods improve crack response and boundary localization in complex backgrounds through attention enhancement or dual-branch structures [3,18,19]; instance/semantic segmentation methods for bridge scenarios (such as the improved SOLOv2 and Enhanced Mask2Former) further combine segmentation results with crack measurement requirements, reflecting the integrated development trend of “segmentation—quantification” [4,14].

In recent years, the application of Transformers in visual tasks has also driven the development of crack segmentation methods. Hierarchical visual Transformers, represented by Swin Transformer, enhance long-range dependency modeling by using windowed attention and cross-window interactions, improving computational efficiency while maintaining performance [16]. Related studies have applied Transformer models to surface crack detection and segmentation on roads and concrete surfaces, showing promising potential in complex backgrounds and multi-scale crack scenarios [10,17]. Additionally, foundational models such as Segment Anything have provided new technical paths for improving crack annotation efficiency, interactive correction, and transfer learning applications [20]. However, the engineering deployment in bridge scenarios still faces challenges such as limited data scale, significant domain shift, and difficulty in maintaining fine crack boundaries.

Apart from network structures, datasets and generalization ability are also key issues in crack segmentation research. Existing studies generally use publicly available datasets such as Crack500 and DeepCrack for training and evaluation, providing unified benchmarks for model comparison [8,9]. However, concrete bridge scenarios differ significantly from road scenes in terms of surface material, lighting conditions, shooting angles, and background interference, meaning the performance of models on public datasets does not necessarily equate to real-world engineering performance [2,3,21]. Some existing work has attempted to enhance cross-scene robustness through data augmentation, deblurring preprocessing, and domain adaptation strategies [21,22,23], but there is still room for improvement in false detection suppression, maintaining the connectivity of fine cracks, and refining boundary delineation in complex bridge backgrounds.

In summary, existing research has provided a rich theoretical and methodological foundation for crack segmentation, but three main shortcomings remain for concrete bridge scenarios: firstly, cracks are similar to interference textures in complex backgrounds, leading to prominent false detection problems; secondly, fine cracks and low-contrast cracks are prone to breakage and missed detection during downsampling and decoding; thirdly, there is still room for improvement in the integrity of segmentation results’ boundaries and their engineering applicability.

Based on the aforementioned motivations, this paper proposes a Boundary-Sensitive Hybrid Attention Network (BSA-Net) for multi-scale crack fine segmentation.

The main contributions of this work are summarized as follows:

1. A hierarchical encoder, Hiera-A, tailored for crack scenarios is proposed, incorporating a lightweight adapter, Adapter, into the backbone to achieve efficient parameter transfer and feature alignment. This approach reduces fine-tuning costs while enhancing the generalization ability from publicly trained domains to engineering domain deployments.
2. A multi-scale feature enhancement module, Light-ASPP, is proposed, which utilizes depthwise separable/dilated convolutions and attention recalibration to achieve multi-receptive field context aggregation. This effectively enhances crack responses and suppresses complex background texture interference.
3. A boundary-aware dual-branch decoding, BAD, is proposed, explicitly injecting high-frequency boundary cues into the segmentation decoding flow. This alleviates edge blurring, breakage, and adhesion issues, achieving fine crack segmentation outputs for engineering scenarios.

## 2. BSA-Net: Fine Crack Segmentation Network Architecture

Let the input image be $I\in R^{H\times W\times 3}$. Due to the high computational cost of image segmentation tasks, and based on current mainstream image segmentation methods, this paper chooses H=W=512. For ease of expression, the feature map size parameters in the following text are all recorded in the form of “height × width × channels.”

### 2.1. Overall Network Architecture

**Figure 1:**
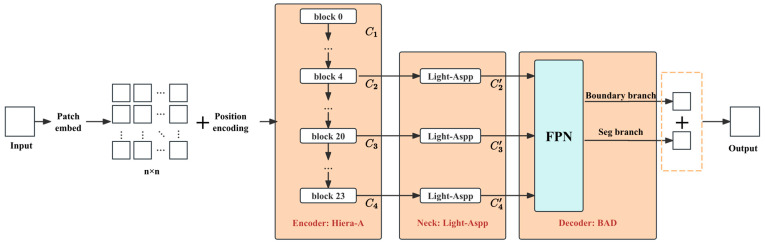
BSA-Net Network architecture.

The overall structure of the proposed crack segmentation network is shown in Figure 1 (overall architecture diagram), which adopts a combination of a hierarchical Transformer encoder, Hiera-A + multi-scale context module, Light-ASPP + boundary-aware decoder, BAD. The core computation flow is summarized as follows:

1. **Patch Embed**: The input image is mapped into a sequence of tokens, and positional encoding is introduced to preserve spatial structure information.
2. **Hiera-A**: Multi-scale features are learned through stacking multi-layer encoding blocks (block) with hierarchical downsampling, producing outputs $\left\{C_{1},C_{2},C_{3},C_{4}\right\}$ (with block 0, block 4, block 20, and block 23 in the diagram representing the outputs of different levels), where features at different scales represent local textures, edge details, and larger semantic contexts. For convenience in subsequent descriptions, the outputs of the encoder at the four scales are denoted as: $C_{1}\in R^{128\times 128\times 112}$, $C_{2}\in R^{64\times 64\times 224}$, $C_{3}\in R^{32\times 32\times 448}$, $C_{4}\in R^{16\times 16\times 896}$. The channel width is then doubled stage by stage. This configuration slightly increases the channel capacity of the hierarchical features and helps preserve more discriminative semantic and boundary cues for fine crack segmentation.
3. **Light-ASPP**: For the multi-scale nature of crack targets, a multi-scale context enhancement is applied to mid-to-high-level features $\left\{C_{2},C_{3},C_{4}\right\}$, producing $\left\{C_{2}^{\prime },C_{3}^{\prime },C_{4}^{\prime }\right\}$ to expand the effective receptive field and preserve fine-grained boundary information.
4. **Boundary-Aware Decoder**: The semantic segmentation main branch (Seg branch) is built using multi-scale fusion based on the Feature Pyramid Network (FPN), while a boundary branch (Boundary branch) is designed to explicitly learn the crack boundary response.
5. **Output Fusion**: The boundary branch and segmentation branch are fused at the output to produce the final crack segmentation result.

### 2.2. Hiera-A Model Based on the Improved SAM2-Hiera Architecture

To address the aforementioned issues, this study proposes a hierarchical visual Transformer model, Hiera-A. The Hiera-A model is built upon the SAM2-Hiera [24] architecture and achieves high-precision feature transfer with minimal computational cost. The overall architecture of Hiera-A follows the “encoder–decoder” paradigm, but with a deep structural reorganization and enhancement of its Backbone.

#### 2.2.1. Hiera-A Backbone Hierarchical Transformer Encoding

**Figure 2:**
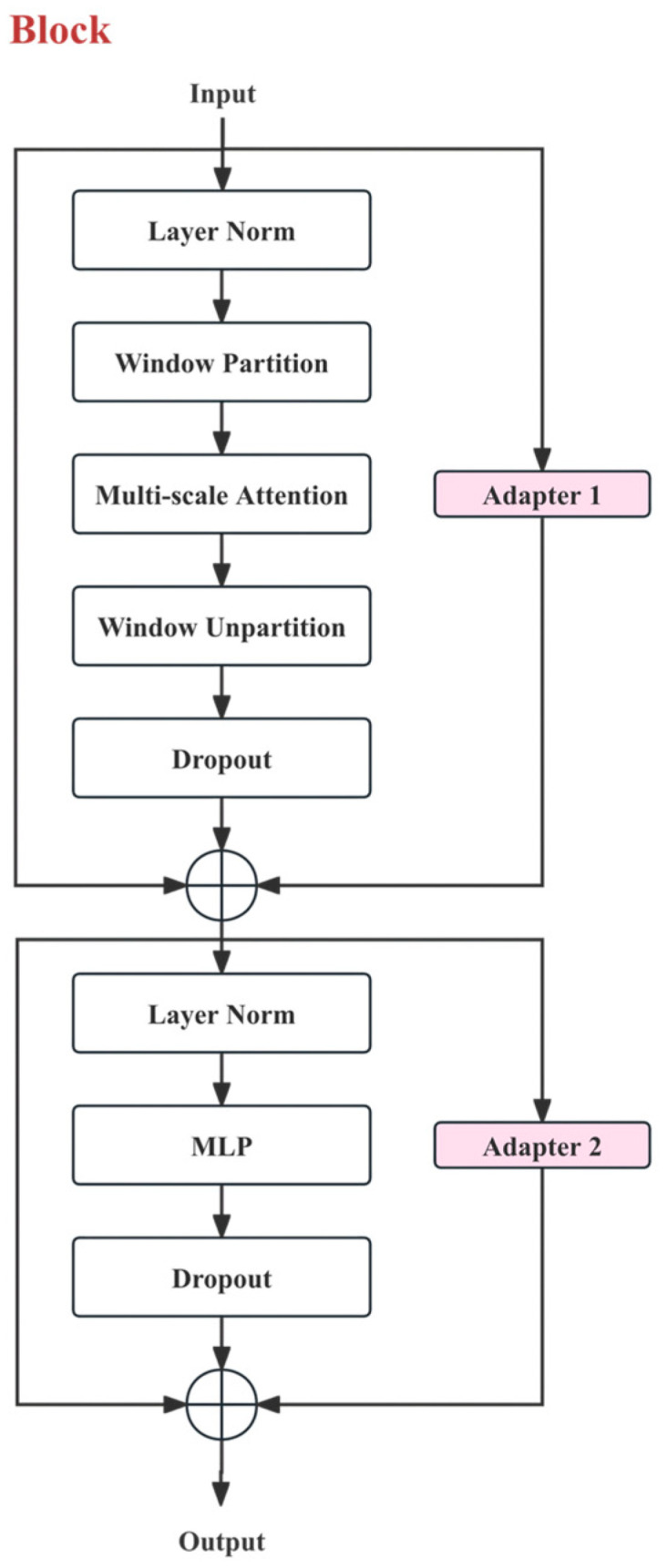
Hiera-A Backbone Network Architecture.

Figure 2 shows the internal structure of a single block. This block adopts the typical “Normalization-Attention/Hybrid-Residual” and “Normalization-MLP-Residual” two-stage structure. Let the input token feature of the block be $X\in R^{N\times d}$, where N=H×W is the number of tokens and d is the channel dimension. The first stage can be written as follows:

(1) $$\tilde{X}=X+\mathrm{Dropout}(A(\mathrm{LN}(X)))+Adapter(X)$$

The second stage is shown below:

(2) $$Y_{1}=\tilde{X}+\mathrm{Dropout}\left(M\left(\mathrm{LN}\left(\tilde{X}\right)\right)\right)+Adapter(\tilde{X})$$

Here, LN(⋅) denotes Layer Normalization; A(⋅) represents the attention/hybrid operator; M(⋅) denotes the two-layer feedforward perceptron MLP; Dropout(⋅) represents regularization with random deactivation to prevent model overfitting; Adapter(⋅) refers to the learnable lightweight adapter.

#### 2.2.2. Hybrid Attention Mechanism

Compared to global self-attention, crack segmentation relies more on local texture continuity and cross-scale context: fine cracks exhibit an elongated topological structure with significant high-frequency boundaries, requiring stable modeling of directional consistency and connectivity within local neighborhoods. Additionally, complex backgrounds introduce strong noise interference, necessitating larger-scale semantic context to suppress false detections. Therefore, this paper adopts a hybrid attention mechanism of “Window Partition/Unpartition + Multi-scale Attention” in the Hiera-A encoding block: first, the features are partitioned into local windows, and multi-scale attention is calculated within the window domain to integrate information from different receptive fields/scales. The window output is then restored to the original spatial layout, with Dropout introduced at the end of the attention branch. This strategy enhances local modeling capabilities while controlling computational complexity, and it also incorporates broader structural information.

The input $X\in R^{N\times d}$ is passed through LayerNorm to obtain $X_{w}=\mathrm{LN}(X)$. Then, Window Partition is applied to divide $X_{w}$ into several local window sets ${\{X}_{w}$}. For any window w, the subset of tokens within the window is represented as:

(3) $$X_{w}\in R^{N_{w}\times d}$$

Here, $N_{w}$ is the number of tokens within the window. Windowing restricts the attention calculation to the local neighborhood, significantly reducing computational complexity and enhancing local structural modeling capabilities.

The goal of multi-scale attention is to introduce information interaction across different receptive fields/scales within the window domain. At a certain scale, the basic form of attention can be written as:

(4) $$A(X_{w})=\mathrm{Softmax}\left(\frac{QK^{T}}{\sqrt{d_{h}}}\right)V$$

(5) $$Q=X_{w}W_{Q},\;K=X_{w}W_{K},\;V=X_{w}W_{V}$$

$W_{Q},W_{K},W_{V}$ are learnable linear mapping matrices, and $d_{h}$ is the dimension of a single head. Thus, the attention output at this scale is:

(6) $$Z\;=\;A(X_{w})V$$

Multi-scale attention is typically achieved by introducing different window scales, varying downsampling ratios, or cross-layer token interactions, enabling the model to provide stable responses to both fine and wide cracks. After aggregating the attention outputs at different scales “Z”, Window Unpartition is used to restore them to the original spatial layout, followed by the introduction of Dropout to improve the generalization stability of the output. This multi-scale mechanism allows the model to preserve high-frequency boundary details of cracks using fine-scale information while leveraging coarse-scale context to enhance overall crack connectivity and suppress false detections from complex background textures, thereby alleviating issues of breakage or local misjudgment caused by a single-scale window.

#### 2.2.3. Lightweight Adapter

There are domain differences between public crack datasets and real engineering images in terms of material, lighting, noise, shooting distance, and crack morphology. If the entire Transformer encoder is fine-tuned, it would require larger memory and longer training time, and it is also prone to overfitting when the data scale is limited. To address this, this paper introduces the parameter-efficient Adapter module within the block, as shown in Adapter 1 and Adapter 2 in Figure 2. This allows domain adaptation and task transfer with a small number of learnable parameters while keeping the backbone parameters relatively stable.

**Figure 3:**
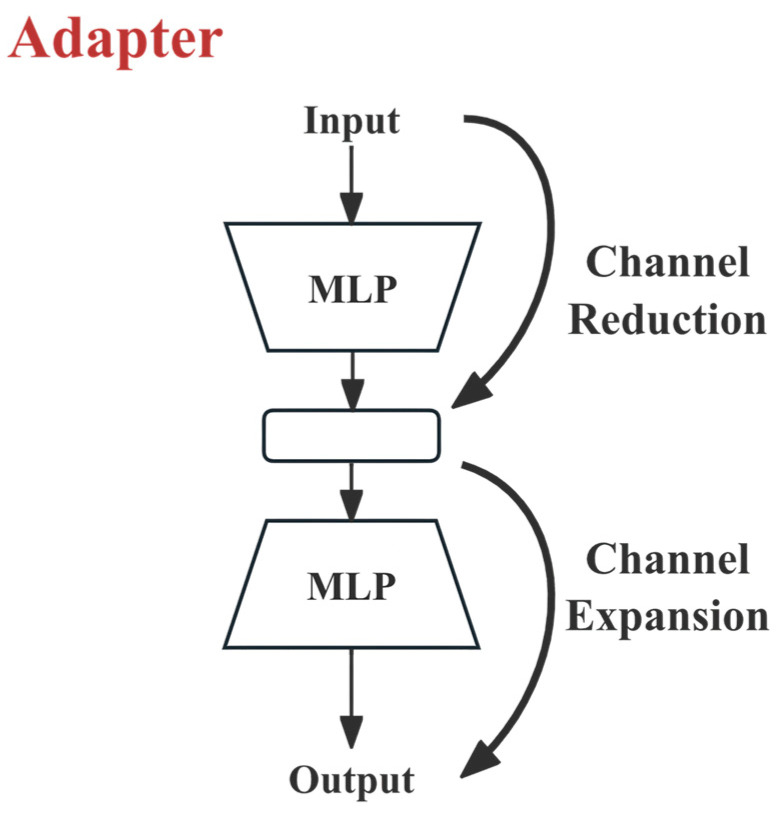
Adapter Network Architecture.

As shown in Figure 3, the Adapter uses a bottleneck structure of “dimension reduction—non-linearity—dimension expansion.” For any input feature $X\in R^{N\times d}$, the mapping of the Adapter is defined as:

(7) $$\mathrm{Adapter}(X)=X+\alpha \cdot f\left(XW_{↓}\right)W_{↑}$$

where $W_{↓}\in R^{d\times r}$ is the dimensionality reduction matrix (r=d/8), which is equivalent to performing 8× downsampling on the input features. For the four encoder stages with channel dimensions (d = 112), 224, 448, and 896, the corresponding bottleneck dimensions are (r = 14), 28, 56, and 112, respectively; $W_{↑}\in R^{r\times d}$ is the dimensionality expansion matrix; f(⋅) is the non-linear activation function GELU; α is the scaling factor, set as a learnable parameter to control the magnitude of the perturbation of the Adapter output on the backbone features.

As shown in Figure 2, Adapter 1 is inserted into the attention branch, and Adapter 2 is inserted into the MLP branch. Their purposes are: (1) to quickly adapt the directionality and cross-scale correlations of cracks during the attention modeling phase; (2) to enhance the separability of crack textures and background textures during the channel mixing phase. Since the parameter scale of the Adapter is proportional to the bottleneck dimension r, this structure improves cross-dataset generalization ability with relatively low additional overhead.

### 2.3. Multi-Scale Feature Enhancement Mechanism

In concrete surface damage detection, particularly for the task of segmenting fine cracks in images, existing deep learning models face a serious “scale-efficiency” paradox. Concrete cracks have an extreme aspect ratio, complex topological structures, and an extremely low foreground-background pixel ratio. To capture subtle cracks that are difficult to detect, models often need to process high-resolution input images, leading to an explosive increase in computational cost.

However, traditional Atrous Spatial Pyramid Pooling (ASPP) modules [25], used for capturing multi-scale contextual information, consume substantial GPU memory and inference time due to their dense dilated convolution operations when processing large-sized feature maps. Moreover, pure convolution operations often lack the ability to filter feature channel importance, leading to confusion between background noise and real crack features.

To address these issues, this paper proposes a multi-scale feature enhancement (Light-ASPP) module as the core Neck component connecting the Backbone and Head. This module integrates multi-receptive field perception, feature fusion, and attention mechanisms in an organic manner. While retaining the advantages of multi-scale context, it achieves context aggregation through a lighter multi-branch dilated convolution and global pooling combination, and introduces channel and spatial attention recalibration to suppress irrelevant responses.

#### 2.3.1. Lightweight Atrous Spatial Pyramid

**Figure 4:**
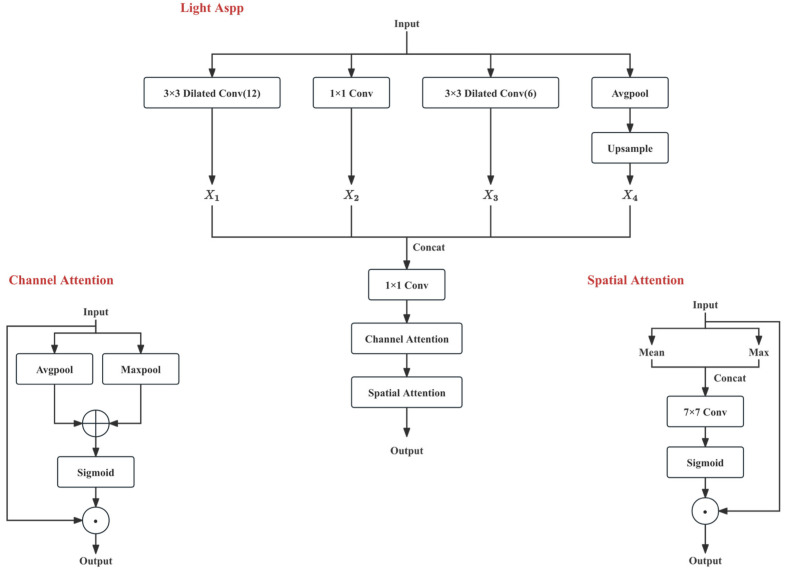
Light-Aspp Network Architecture.

As shown in Figure 4, the input feature is denoted as $Y_{1}\in R^{h\times w\times c}$. The module consists of four parallel branches:

- Branch 1: 3 × 3 Dilated Convolution with dilation rate d=12, output $X_{1}$;
- Branch 2: 1 × 1 Convolution, output $X_{2}$;
- Branch 3: 3 × 3 Dilated Convolution with dilation rate d=6, output $X_{3}$;
- Branch 4: Global Average Pooling followed by Upsampling to h×w, output $X_{4}$.

The output of each branch is h×w×c/4. The effective receptive field of the dilated convolution can be expressed as:

(8) $$k_{\mathrm{eff}}=k+(k-1)(d-1)$$

k is the size of the convolutional kernel. This value denotes the effective kernel size of one dilated convolution layer, rather than the cumulative receptive field measured on the original input image after multiple network layers. The overall input-pixel receptive field depends on the full hierarchy, including previous convolutions, strides, and downsampling operations. By setting different dilation rates, the module achieves a larger contextual range without significantly increasing the number of parameters. This helps in semantic completion across crack fracture regions and suppresses local misjudgments caused by texture noise.

With a 3 × 3 kernel, the dilation rates (d = 6) and (d = 12) correspond to effective kernel sizes of 13 and 25, respectively. These two branches provide complementary medium- and large-scale contextual ranges. The (d = 6) branch helps model local-to-medium crack continuity, while the (d = 12) branch captures larger structural context. This is useful for recovering discontinuous cracks and suppressing texture-induced false positives without increasing the kernel size.

The outputs of the four branches are concatenated along the channel dimension, where Concat(⋅) denotes the concatenation operation:

(9) $$X_{C}=\mathrm{Concat}(X_{1},X_{2},X_{3},X_{4})$$

#### 2.3.2. Fusion and Dimensionality Reduction

After the feature maps from each branch are aggregated through concatenation, this paper introduces a 1 × 1 convolution immediately after the concatenation layer:

**Cross-Channel Information Interaction**: Only after the feature fusion from different branches can the information captured by different receptive fields (such as local textures and global lighting) interact. The 1 × 1 Convolution achieves non-linear fusion of multi-scale contexts through linear combinations. The mathematical expression is:

(10) $$X_{F}={\mathrm{Conv}}_{1\times 1}(X_{C})$$

#### 2.3.3. Feature Refinement Based on Channel and Spatial Attention Channel Attention and Spatial Attention

To further highlight the crack response and suppress background textures, Light-ASPP sequentially introduces Channel Attention and Spatial Attention after fusion.

1. **Channel Attention**: Perform Global Average Pooling and Max Pooling on $X_{F}$ along the spatial dimension to obtain a c-dimensional descriptor:

(11) $$z_{\mathrm{avg}}=\mathrm{GAP}(X_{F}),\;z_{\max}=\mathrm{GMP}(X_{F})$$

Both are passed through a shared MLP mapping and summed, then passed through a Sigmoid activation function to obtain the channel weight vector:

(12) $$s_{c}=\sigma (\mathrm{MLP}(z_{\mathrm{avg}})+\mathrm{MLP}(z_{\max}))$$

Finally, channel recalibration is performed to output:

(13) $$X_{CAT}=X_{F}⊙s_{c}$$

Here, ⊙ denotes element-wise multiplication along the channel dimension.

2. **Spatial Attention**: Perform mean and max aggregation on $X_{\mathrm{CAT}}$ along the channel dimension:

(14) $$m_{\mathrm{avg}}={\mathrm{Mean}}_{c}\left(X_{CAT}\right),\;m_{\max}={\mathrm{Max}}_{c}(X_{CAT})$$

After concatenation, apply a 7 × 7 Convolution followed by a Sigmoid activation to obtain the spatial weight map:

(15) $$s_{s}=\sigma ({\mathrm{Conv}}_{7\times 7}(\mathrm{Concat}(m_{\mathrm{avg}},m_{\max})))$$

Finally, perform spatial recalibration to output:

(16) $$Y_{2}=X_{CAT}⊙s_{s}$$

By setting different dilation rates, the module can simultaneously cover local texture and larger structural information, helping to compensate for local fractures and suppress background texture interference. After concatenating the multi-branch outputs along the channel dimension, a 1 × 1 convolution is applied to fuse them and obtain enhanced features. To further highlight the crack regions, the module chains channel attention and spatial attention: channel attention emphasizes the filter responses related to crack textures, while spatial attention highlights the elongated structural areas at the pixel level, thereby improving segmentation stability under weak contrast and complex background conditions.

### 2.4. Boundary-Aware Dual-Branch Decoder

In the task of concrete surface crack detection, especially for fine crack targets in large-size images, traditional encoder–decoder architectures often face severe challenges. Cracks typically appear as extremely fine linear topological structures with a very low pixel ratio, often submerged in complex background textures. Cracks have different feature distribution patterns between the interior and the boundary: the interior is relatively smooth, while there are sharp gradient changes at the boundary. Existing semantic segmentation networks, during deep feature extraction, are able to capture high-level semantic information. However, as the downsampling ratio increases, high-frequency spatial details are inevitably lost through pooling operations. Forcing the learning of these two types of features in a single branch leads to optimization conflicts, resulting in blurry, broken, or fused masks at the crack boundaries during the decoding stage.

**Figure 5:**
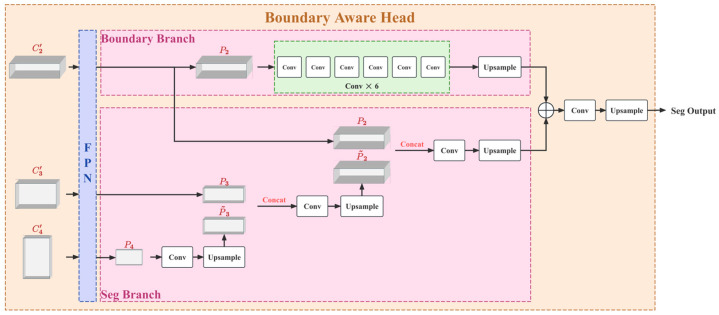
BAD Network Architecture.

To address these issues and significantly enhance the ability to capture the boundaries of fine cracks, this paper proposes an innovative decoder structure—the Boundary-Aware Dual-Branch Decoder (BAD). This module aims to explicitly model the boundaries and interact features across two streams, greatly retaining and correcting boundary information while restoring image resolution. As shown in Figure 5, BAD consists of three core components:

1. A multi-scale feature aggregation module based on FPN [26], designed to alleviate the semantic gap caused by scale variations;
2. A parallel dual-branch processing architecture, responsible for main semantic segmentation and high-frequency boundary prediction, defined as the Segmentation Branch and the Boundary Branch;
3. Dual-branch fusion, where residual connections inject explicit boundary cues into the segmentation flow, enabling pixel-level prediction with refined boundaries.

#### 2.4.1. Multi-Scale Feature Pyramid

Cracks in images often exhibit multi-scale characteristics, ranging from visible primary cracks to micron-level microcracks. A single-scale feature map is insufficient to simultaneously capture global context information and local detail features. Therefore, BAD first uses FPN as a bridge between the Neck and Head.

The feature map set output by the Backbone at four different stages is $\left\{C_{1},C_{2},C_{3},C_{4}\right\}$. For the fine features of cracks, this paper only uses the mid-to-high-level features $\left\{C_{2},C_{3},C_{4}\right\}$. After passing through Light-ASPP, they are represented as $\left\{C_{2}^{\prime },C_{3}^{\prime },C_{4}^{\prime }\right\}$, with downsampling steps relative to the input image of {8, 16, 32}. To construct a feature pyramid with strong semantics and high resolution, this paper adopts a top-down path and lateral connection strategy.

Specifically, the top-level feature $C_{4}^{\prime }$ is first passed through a 1 × 1 Convolution to reduce the number of channels to 256, resulting in $P_{4}^{\prime }$. Then, for all aggregated feature layers with i∈{3,2}, the aggregated feature $P_{i}$ at layer i is calculated as follows:

(17) $$P_{i}={\mathrm{Conv}}_{1\times 1}(C_{i}^{\prime })+{\mathrm{Upsample}}_{2\times }(P_{i+1}^{\prime })$$

Here, ${\mathrm{Upsample}}_{2\times }$ denotes the bilinear interpolation upsampling operation, and ${\mathrm{Conv}}_{1\times 1}$ is used to align the number of channels. To eliminate the aliasing effects generated during the upsampling process, a 3 × 3 Convolution is further applied to each fused $P_{i}^{\prime }$, resulting in the final pyramid feature set $\left\{P_{2},P_{3},P_{4}\right\}$. This process not only effectively aggregates the abstract semantics of deep features but also reuses shallow spatial positional information through lateral connections, providing a rich information foundation for the subsequent dual-branch decoding.

#### 2.4.2. Segmentation Branch

As shown in Figure 5, the features from FPN are $\left\{P_{2},P_{3},P_{4}\right\}$, with resolutions corresponding to 64 × 64, 32 × 32, and 16 × 16, and all having 256 channels. The segmentation branch starts with the lowest resolution $P_{4}$, and proceeds as follows:

1. $P_{4}$ is fused through convolution and upsampled to obtain ${\tilde{P}}_{3}$, with a size of 32 × 32 × 128;
2. ${\tilde{P}}_{3}$ is concatenated with $P_{3}$, then fused through convolution and upsampled to obtain ${\tilde{P}}_{2}$, with a size of 64 × 64 × 64;
3. ${\tilde{P}}_{2}$ is concatenated with $P_{2}$, and fused through convolution to obtain the segmentation feature $X_{S}$, with a size of 128 × 128 × 32.

In formula form, it can be written as:

(18) $${\tilde{P}}_{3}={\mathrm{Upsample}}_{2\times }(\psi_{4}(P_{4}))$$

(19) $${\tilde{P}}_{2}={\mathrm{Upsample}}_{2\times }\left(\psi_{3}\left(\mathrm{Concat}\left(P_{3},{\tilde{P}}_{3}\right)\right)\right)$$

(20) $$X_{S}=\psi_{2}\left(\mathrm{Concat}\left(P_{2},{\tilde{P}}_{2}\right)\right)$$

Here, $\psi_{k}\left(\cdot \right)$ denotes the convolution fusion operator, which is implemented as convolution-batch normalization-activation (Conv-BN-Activation).

#### 2.4.3. Boundary Branch

The main errors in crack segmentation are often concentrated around the boundaries, and fine cracks are prone to breakage and missed detection. To address this, this study introduces a boundary branch to explicitly regress the boundary response, with the structure shown in the upper part of Figure 5: The boundary branch takes the highest-resolution feature $P_{2}$ as input, extracts boundary-sensitive features through multiple layers of convolutional stacking, and then applies convolution and upsampling to obtain boundary-guided features $X_{B}$, with a size of 128 × 128 × 32. The core purpose of this branch is to: enhance the precision of crack boundary localization by emphasizing the grayscale-texture transition regions on both sides of the crack, and correct for fine crack regions when fused with the main branch in subsequent stages.

The boundary branch can be abstracted as:

(21) $$X_{B}={\mathrm{Upsample}}_{2\times }\left(\eta (\;P_{2}\;)\right)$$

Here, η(⋅) is the boundary feature extraction network, composed of 6 convolutional layers.

#### 2.4.4. Dual-Branch Fusion

At the output stage, the boundary features are fused with the segmentation features through addition, followed by convolution and upsampling to obtain the final crack probability map, with a size of 512 × 512 × 2, representing the binarized segmentation result:

(22) $$Y_{3}=X_{S}⊕X_{B}$$

(23) $$Y_{\mathrm{out}}={\mathrm{Upsample}}_{2\times }\left(\mathrm{Conv}\left(Y_{3}\right)\right)$$

Here, ⊕ denotes element-wise addition.

### 2.5. Loss Function and Training Objective

Crack segmentation exhibits distinct statistical characteristics at the pixel level. Specifically, foreground cracks usually appear as elongated and slender structures, occupying a substantially smaller area than the background concrete regions. Meanwhile, background disturbances such as surface voids, mortar textures, shadows, and contamination on concrete surfaces may introduce a large number of hard-to-classify samples. Under such circumstances, the use of a single loss function tends to induce two typical biases during training. The first is a background-dominated minimization bias. When the proportion of positive samples is extremely low, the gradient of the averaged loss is mainly contributed by background pixels, causing the model to suppress overall false positives at the expense of crack recall. The second is insufficient learning of hard samples, whereby boundary transition regions, low-contrast fine cracks, and complex-texture areas fail to receive adequate attention.

To address these issues, in conjunction with the boundary-aware dual-branch decoder, this study further introduces boundary supervision on the basis of region-level segmentation supervision. Accordingly, a joint optimization objective consisting of “region segmentation loss + boundary constraint loss” is established. The main segmentation branch is designed to learn the overall semantic consistency of crack regions, whereas the boundary branch specifically captures high-frequency variation information around crack contours. Through joint backpropagation and boundary feature feedback, the proposed objective jointly enhances the model’s localization capability for fine-grained edges and its robustness against complex background interference.

#### 2.5.1. Joint Supervision Modeling

Let the training sample be the input image $I\in R^{H\times W\times 3}$, and let the pixel-level annotation be $Y\in \{0,1{\}}^{H\times W}$, where $Y_{i}=1$ indicates a crack foreground pixel and $Y_{i}=0$ indicates a background pixel.

For the segmentation branch, let the network output be defined as the foreground probability map of cracks, which is expressed as:

(24) $$P^{seg}=f_{seg}(I;\theta )\in [0,1{]}^{H\times W},\;\;\;P_{i}^{seg}=\sigma (z_{i}^{seg})$$

where $f_{seg}(\cdot ;\theta )$ denotes the segmentation model parameterized by *θ*; $z_{i}^{seg}$ represents the logit classification score of pixel *i*; and *σ*(·) is the Sigmoid activation function. The learning objective is to optimize *θ* on the training set $D=\{(I_{k},Y_{k}){\}}_{k=1}^{N}$, such that the model output $P^{seg}$ approaches the ground-truth annotation *Y* as closely as possible in terms of both region overlap and boundary consistency, while maintaining robustness under complex scene interference. In this study, the region segmentation loss is formulated as a combination of multiple complementary loss terms, thereby constraining the learning process from different perspectives.

For the boundary branch, let its output is defined as the boundary probability map $P^{bnd\;}$ generated from the highest-resolution feature of the FPN, expressed as:

(25) $$P^{bnd}=f_{bnd}(P_{2};\theta )\in [0,1{]}^{H_{b}\times W_{b}},P_{j}^{bnd}=\sigma (z_{j}^{bnd})$$

where $H_{b}\times W_{b}$ denotes the prediction size of the boundary branch. Since the boundary branch directly operates on higher-resolution feature maps, it is more suitable for characterizing local abrupt responses around crack contours.

Therefore, the training objective of this study is no longer limited to enforcing consistency between the predicted region mask and the ground-truth annotation. Instead, optimization is simultaneously performed from three perspectives: region overlap, hard-sample focusing, and boundary alignment. This design aims to improve the fine-grained segmentation capability of the model in complex bridge underside surface scenarios.

#### 2.5.2. Region Segmentation Loss

To ensure class discrimination capability, robustness to class imbalance, and the ability to focus on hard samples, this section constructs the region segmentation loss $L_{Region}$ as a weighted combination of cross-entropy loss (CE Loss), Dice loss, and Focal loss. The cross-entropy loss is based on pixel-wise negative log-likelihood, providing stable and direct classification gradients and ensuring overall discriminative ability and convergence stability. The Dice loss measures the consistency between prediction and annotation from the perspective of set overlap, thereby significantly mitigating the bias caused by foreground scarcity. The Focal loss assigns larger weights to hard-to-classify pixels, suppressing the gradient domination effect caused by a large number of easily classified background pixels and strengthening the learning of fine cracks and low-contrast regions.

Based on the complementary properties of the three, the region segmentation loss is defined as:

(26) $$L_{Region}=L_{CE}+L_{Dice}+0.5L_{Focal}$$

#### 2.5.3. Boundary Branch Loss

Compared with regional foreground pixels, boundary pixels are more sparsely distributed, and their supervision signals are more easily overwhelmed by a large number of non-boundary pixels during training. To enhance the model’s perception of crack contours, this study supervises the boundary branch using BCEWithLogitsLoss with positive-sample weighting.

Let the target boundary map generated from the boundary annotation be $B\in \{0,1{\}}^{H\times W}$. Since the prediction resolution of the boundary branch is $H_{b}\times W_{b}$, B first needs to be downsampled to match the size of the boundary prediction, expressed as:

(27) $$B^{↓}=D\left(B\right)\in \{0,1{\}}^{H_{b}\times W_{b}}$$

where *D*(·) denotes the size-alignment operation. To preserve the discreteness and geometric contour characteristics of the boundary labels as much as possible, a label transformation strategy consistent with the prediction size is adopted for alignment in practice. The boundary loss is defined as:

(28) $$L_{bnd}=-\frac{1}{N_{b}}\sum_{j=1}^{N_{b}}\left[w_{pos}B_{j}^{↓}ln\left(P_{j}^{bnd}\right)+\left(1-B_{j}^{↓}\right)ln\left(1-P_{j}^{bnd}\right)\right]$$

where $N_{b}=H_{b}\times W_{b}$, and $w_{pos}$ is the positive boundary sample weight, which is set to 10 in this study. This compensates for the supervision imbalance caused by the fact that boundary positive samples are far fewer than non-boundary pixels. The design significantly strengthens the model’s response to crack contours, endpoints, and local discontinuities, thereby providing clearer edge-guidance information for the subsequent segmentation branch.

#### 2.5.4. Boundary Label Generation

To avoid the additional annotation cost associated with manually labeling boundaries, this study does not construct separate manual boundary annotations. Instead, boundary supervision labels are generated online directly from the segmentation masks. Specifically, the segmentation annotation *Y* is first binarized; subsequently, a morphological gradient operator is employed to extract the edge region, thereby obtaining the boundary label, expressed as:

(29) B=Dilate(Y)−Erode(Y)

where *Dilate*(·) and *Erode*(·) denote dilation and erosion operations, respectively. In this study, a structural element with width = 1 is adopted to obtain a narrow and well-localized boundary response region. This region approximates the contour transition bands on both sides of the crack mask and provides supervision consistent with the high-frequency structures targeted by the boundary branch.

During data preprocessing, the generated boundary labels participate in data augmentation operations such as random flipping, scaling, and cropping together with the input images and segmentation masks, and are then uniformly converted into tensors as network inputs. This ensures strict spatial alignment among the image content, region mask, and boundary supervision, thereby avoiding pseudo-supervision errors caused by inconsistent geometric transformations. Since the boundary labels are entirely generated online from the existing masks, this mechanism does not introduce any additional manual annotation burden. Meanwhile, it can dynamically adapt to sample augmentation during training, demonstrating favorable implementation simplicity and engineering reproducibility.

#### 2.5.5. Joint Loss Function

Based on region supervision and boundary supervision, the final joint loss function in this study is defined as:

(30) $$L_{total}=L_{seg}+L_{bnd}$$

## 3. Experimental Design and Result Analysis

### 3.1. Experimental Environment Configuration

To comprehensively validate the effectiveness and robustness of the proposed BSA-Net model in the multi-scale crack fine segmentation task, systematic comparison experiments and ablation studies were conducted under a unified experimental environment and data configuration. This section provides a detailed explanation of the experimental hardware environment and software configuration to ensure the reproducibility and fairness of the experimental results.

All experiments in this paper were conducted in the same hardware and software environment to eliminate the impact of platform differences on the experimental results. The hardware and software configurations used in the experiments are consistent with those in Table 1. During the training process, all models were trained with a single GPU, and the network parameters were initialized with the same random seed to ensure comparability between different experiments.

To ensure fairness in the training of the comparison methods and the reproducibility of the results, the AdamW optimizer was uniformly used to perform end-to-end optimization of the network parameters. The input resolution was set to 512 × 512, with an initial learning rate of $1\times 10^{-4}$ and a weight decay coefficient of $1\times 10^{-4}$. The total number of training epochs was 150, with a batch size of 8. Other than differences in network structure, the remaining training configurations were kept consistent across all methods. The learning rate schedule used the Poly strategy, with a polynomial decay exponent of p=0.9. This scheduling strategy maintains a larger update step size in the early training stages to accelerate convergence and gradually decreases the learning rate in the later stages to refine the parameter search, which helps improve the connectivity and boundary alignment accuracy of segmentation results in fine crack regions. All models were trained until convergence, with the number of training epochs remaining consistent, and the best-performing model on the validation set was selected for subsequent testing. Additionally, the region segmentation joint loss, as proposed in Section 2.5, was used for end-to-end training.

### 3.2. Dataset Composition

To thoroughly evaluate the robustness and generalization ability of the BSA-Net model, three publicly available pixel-level annotated datasets were selected: Crack500 [27], DeepCrack [28], and GAPs384 [29]. These datasets differ significantly in terms of acquisition devices, resolution, background complexity, crack morphology, and annotation styles, and can be used to create more representative training distributions, allowing for a stricter evaluation of the model’s generalization ability.

To ensure the objectivity and stability of the experimental results, the training, validation, and test sets of each dataset are kept non-overlapping, with a ratio of 7:2:1. Input images are randomly cropped to 512 × 512 pixels to ensure the test results accurately reflect the model’s generalization ability in unseen scenarios. The validation set is used for both early stopping and model selection, while the test set is used only for final evaluation. During training, the validation performance is monitored after each epoch. If the validation metric does not improve for 10 consecutive epochs, the training process is stopped early. The checkpoint with the best validation performance is selected as the final model and evaluated on the test set.

To enhance the credibility and reproducibility of the experimental results, a fixed random seed is used to control the data loading order and network initialization process in all experiments. Additionally, all comparison models use the same data preprocessing pipeline, training strategy, and evaluation methods, ensuring that the experimental comparisons reflect only the differences in model structures and methodologies.

### 3.3. Evaluation Metrics

Considering the characteristics of crack detection tasks, such as multiple target scales, elongated shapes, and the high cost of omissions, this paper uses various evaluation metrics to comprehensively assess model performance from different perspectives.

1. mean Intersection over Union (mIoU).
2. Precision and Recall.
3. $F_{1}$ Score: The $F_{1}$ score is an evaluation metric for binary classification tasks. Under the premise that positive and negative samples are defined consistently in the binary classification task, the $F_{1}$ score is mathematically equivalent to the Dice coefficient introduced in Section 2.5. It combines both precision and recall. The $F_{1}$ score is a single value that can be used to measure the performance of a classifier.
  (31) $$F_{1}=\frac{2\cdot Precision\cdot Recall}{Precision+Recall}$$
4. HD95: The 95% Hausdorff Distance (HD95) is an evaluation metric for measuring the boundary accuracy of image segmentation results. In pixel-level segmentation tasks, it can be adopted to quantify the degree of spatial deviation between predicted boundaries and ground-truth boundaries.

### 3.4. Overall Performance Evaluation of BSA-Net

In this section, the overall performance of the proposed BSA-Net for multi-scale fine crack segmentation is evaluated through comparative experiments with several mainstream image segmentation methods from both quantitative and qualitative perspectives. All experiments were conducted under the unified experimental settings described in Section 3.1 and were assessed using the evaluation metrics defined in Section 3.3.

A series of representative image segmentation methods were selected as baseline models, and their results were analyzed using multiple evaluation metrics in both quantitative and qualitative terms. To avoid one-sided comparisons, the compared methods included SegFormer [30], U-Net [11], PSPNet [31], DeepLabV3 [13], BiSeNet [32], DANet [33], and OCRNet [34].

All comparison methods were trained and evaluated using the same training, validation, and test set partitions to ensure the fairness and comparability of the experimental results.

#### 3.4.1. Quantitative Comparison of Evaluation Metrics

**Figure 6:**
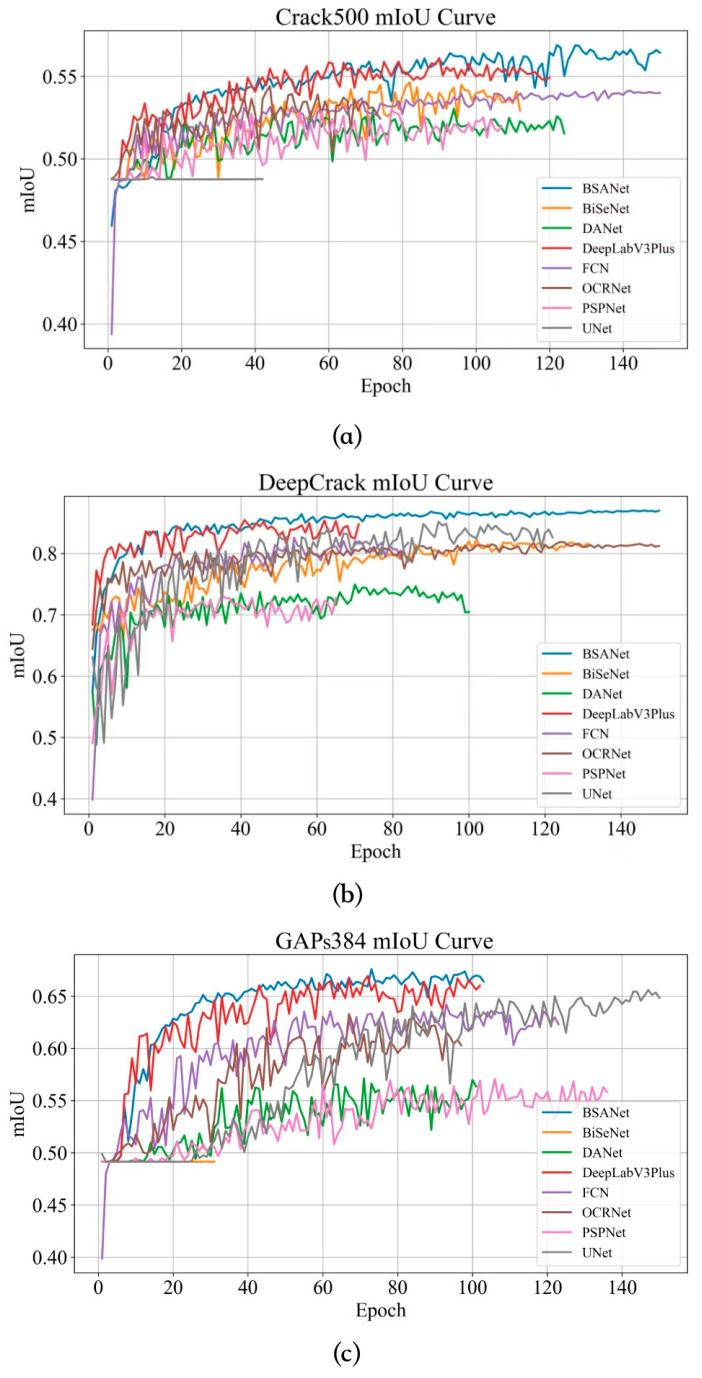
The mIoU curves of BSA-Net and the various models on three datasets: (**a**) Crack500 dataset; (**b**) DeepCrack dataset; (**c**) GAPs384 dataset.

Table 2, Table 3 and Table 4 summarize the evaluation results of BSA-Net and the competing methods on each dataset, including mIoU, Precision, Recall, Dice, and $F_{1}$ score. Figure 6 illustrates the variation in mIoU with increasing epochs for BSA-Net and the compared methods across different datasets. In the figure, SegFormer, U-Net, PSPNet, DeepLabv3+, BiSeNet, DANet, OCRNet, and BSA-Net are represented by the pink, gray, brown, red, orange, green, purple, and blue curves, respectively.

Next, the performance of each model is analyzed on a dataset-by-dataset basis.

1. Crack500 Dataset

On the Crack500 dataset, all models generally exhibit moderate mIoU, low Recall, and large HD95, indicating that the combined effects of complex textured backgrounds, low-contrast fine cracks and severe class imbalance in this dataset collectively increase the difficulty of crack segmentation. Specifically, the proposed BSA-Net achieves an mIoU of 0.589, a Recall of 0.308, an $F_{1}$ score of 0.343, and an HD95 of 181.37. Taking DeepLabV3+, the comparison model with the optimal mIoU performance, as the reference, BSA-Net improves mIoU from 0.558 to 0.589, representing a relative improvement of 5.56%. Taking OCRNet, the comparison model with the optimal $F_{1}$ score, as the reference, BSA-Net increases the $F_{1}$ score from 0.251 to 0.343, a relative improvement of 36.65%. Taking DeepLabV3+, the comparison model with the optimal Recall performance, as the reference, BSA-Net raises Recall from 0.181 to 0.308, corresponding to a 70.17% relative improvement. Meanwhile, in terms of boundary localization accuracy, with DeepLabV3+, the comparison model with the optimal HD95 performance, as the reference, BSA-Net reduces HD95 from 257.24 to 181.37, a relative reduction of 29.49%, indicating that the spatial deviation between the predicted boundary and the ground-truth boundary is significantly reduced. Although BSA-Net does not achieve the highest Precision on this dataset: its Precision is 0.388, which is slightly lower than the 0.390 of DeepLabV3+ (only a 0.51% relative decrease) and also lower than the 0.421 of OCRNet, it still shows more obvious advantages in mIoU, Recall, $F_{1}$ score and HD95 overall. This demonstrates that under the condition of coexistence of complex textured backgrounds and fine cracks, the advantage of BSA-Net does not lie in reducing false detections through conservative prediction, but in that it can significantly improve the continuous detection capability, regional coverage consistency and boundary fitting degree of fine and weak cracks while maintaining relatively stable Precision.

2. DeepCrack Dataset

The overall performance metrics on the DeepCrack dataset are significantly superior to those on the Crack500 dataset, which indicates that the DeepCrack dataset features clearer crack structures, higher annotation consistency, relatively weaker background interference, and model differences are mainly reflected in the capabilities of boundary delineation and false detection suppression. Among all tested models, BSA-Net achieves the highest mIoU of 0.866; compared with the best-performing baseline model SegFormer whose mIoU is 0.859, BSA-Net obtains an improvement of 0.82%; in comparison with DeepLabV3+ which yields an mIoU of 0.858, the improvement reaches 0.93%. Meanwhile, BSA-Net also achieves the highest Precision of 0.924 across all models, representing an increase of 1.32% compared with U-Net (0.912) and an increase of 3.70% compared with DeepLabV3+ (0.891). In terms of the boundary error metric, BSA-Net obtains an HD95 of 16.07, which is 2.84% lower than DeepLabV3+ (16.54) and 23.07% lower than SegFormer (20.89), demonstrating its superior performance in boundary localization and contour alignment. However, the Recall of BSA-Net is 0.774, which is 3.73% lower than U-Net (0.804) and 3.37% lower than DeepLabV3+ (0.801); its $F_{1}$ score is 0.842, which is almost on par with DeepLabV3+, but is 1.17% slightly lower than U-Net (0.854). This result indicates that the advantage of BSA-Net on this dataset does not mainly originate from expanding the foreground region to obtain a higher Recall, but instead achieves higher Precision, lower boundary error and the highest overall regional overlap through stricter boundary constraints and stronger background suppression capability. Compared with the Crack500 dataset, the DeepCrack dataset has clearer crack structures and higher annotation consistency; therefore, the high Precision and low HD95 exhibited by BSA-Net in this scenario can better reflect its advantages in boundary restraint, false detection suppression and result reliability.

3. GAPs384 Dataset

On the GAPs384 dataset, the performance gap among all models further expands, indicating that this dataset contains more prominent real-world road textures, illumination variations and non-crack linear interferences, making models more prone to such issues as false detection expansion, boundary dislocation and foreground collapse. Among all tested models, BSA-Net achieves the strongest comprehensive performance. Taking DeepLabV3+, the comparative model with the optimal overall performance, as the reference, the mIoU of BSA-Net increases from 0.673 to 0.684, with a relative improvement of 1.63%; the Recall rate increases from 0.496 to 0.564, with a relative improvement of 13.71%; the $F_{1}$ score increases from 0.540 to 0.577, with a relative improvement of 6.85%; and the HD95 decreases from 61.32 to 58.48, with a relative reduction of 4.63%. This demonstrates that BSA-Net can not only improve the detection completeness of crack regions, but also further reduce the boundary deviation between predicted contours and ground-truth contours. Although its Precision is 0.592, which is only 0.17% lower than the 0.593 of DeepLabV3+ and not the highest, and it is 7.79% lower than U-Net, the model with the highest Precision, this difference does not weaken the overall optimal performance of BSA-Net. On the contrary, on the premise that the Precision remains basically stable, BSA-Net significantly improves the Recall and $F_{1}$ score while synchronously reducing HD95, which indicates that in complex texture backgrounds, strong interference and cross-domain scenarios, BSA-Net can track crack bodies and discontinuous crack segments more stably, and maintain higher regional consistency and boundary accuracy. For real-world bridge disease inspection scenarios, this capability of improving Recall and improving boundary positioning without excessively sacrificing Precision has higher engineering application value.

For a fair and controlled comparison, all baseline methods and the proposed BSA-Net were trained and evaluated under the same experimental settings, including dataset split, input resolution, preprocessing, data augmentation, optimizer, learning rate schedule, batch size, maximum training epochs, early stopping criterion, and evaluation metrics. The checkpoint with the best validation performance was selected for testing. It should be noted that this unified protocol aims to ensure comparability among different methods, but it may not represent the individually optimal hyperparameter configuration for each baseline model. In our experiments, several baselines, such as U-Net on Crack500 and BiSeNet on GAPs384, exhibited unstable convergence under the unified setting, which may explain their relatively weak results.

In summary, the advantages of BSA-Net should be comprehensively understood from the task priorities of different datasets: On Crack500 and GAPs384, two datasets featuring complex backgrounds, thin and faint cracks that are prone to discontinuity, the advantages of BSA-Net are mainly reflected in the simultaneous improvement of Recall, mIoU, $F_{1}$ score and HD95, indicating that the model can reduce missed detection while improving the spatial fitting degree of crack boundaries. On datasets with relatively clear crack structures such as DeepCrack, the advantages of BSA-Net are mainly reflected in higher Precision and mIoU, as well as lower HD95, demonstrating that its output has greater advantages in boundary purity, false detection suppression and contour localization accuracy. It can thus be concluded that the BSA-Net proposed in this paper not only improves the overall performance of crack region segmentation, but also significantly enhances the model’s ability to recover and constrain fine-grained boundary structures, thereby achieving higher comprehensive segmentation quality in a variety of complex scenarios.

#### 3.4.2. Visual Comparison

**Figure 7:**
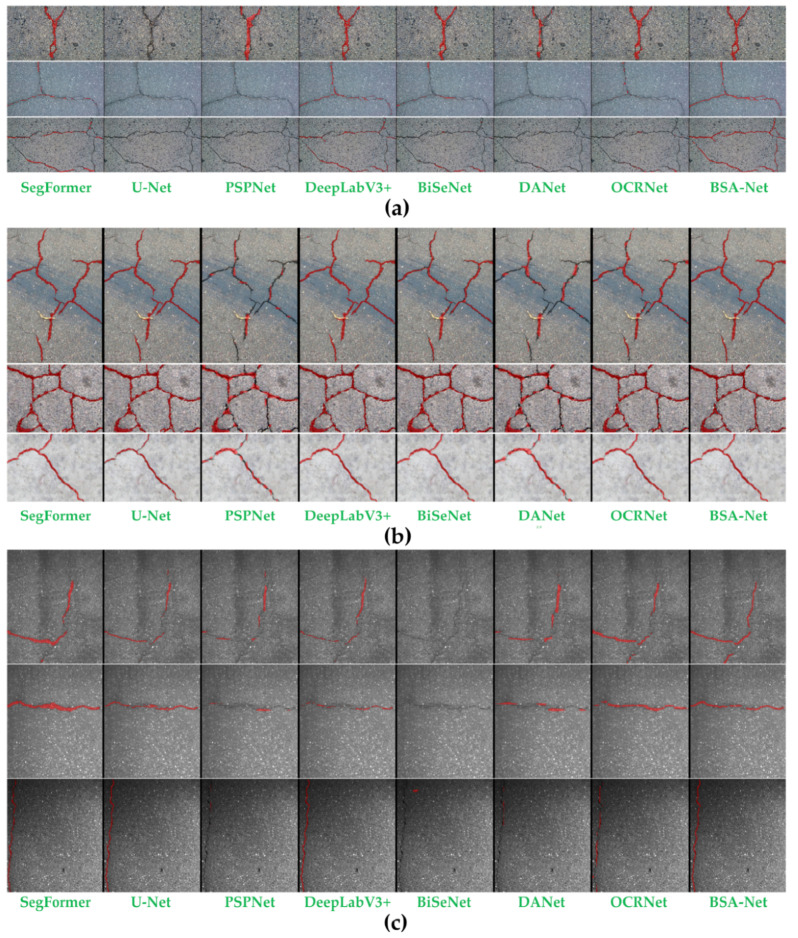
Visual comparison of BSA-Net with the various models on three datasets: (**a**) Crack500 dataset; (**b**) DeepCrack dataset; (**c**) GAPs384 dataset.

To further investigate the error patterns and discriminative behaviors of different semantic segmentation methods in elongated crack scenarios, three representative sample groups from the three datasets were selected for visual comparison, as shown in Figure 7. From left to right, the segmentation results of SegFormer, U-Net, PSPNet, DeepLabV3+, BiSeNet, DANet, OCRNet, and BSA-Net are presented, respectively. The red regions denote the crack foreground predicted by each model.

The advantages of BSA-Net over three public datasets are manifested as follows: in scenarios of varying complexity, BSA-Net can respectively enhance crack detection capability, boundary suppression capability and overall consistency in accordance with task requirements, thereby forming a more stable overall advantage. For the crack segmentation task, which features slender structures, extremely imbalanced classes and significant background interference, BSA-Net establishes an indicator balancing method that better meets engineering requirements.

The imaging characteristics of the Crack500 dataset include narrow crack width, weak grayscale contrast, complex background texture, and a large number of pseudo-edges with similar morphology to cracks, which imposes higher requirements on the model’s fine-grained representation capability, boundary preservation capability and false detection suppression capability. In the DeepCrack dataset, crack morphology is generally clearer, the grayscale and texture contrast between cracks and the background is stronger, and crack connectivity and boundary distinguishability are better, so the overall performance of all models on this dataset is generally higher. However, its samples still contain typical challenges such as bifurcated cracks, intersecting cracks, locally weak-texture crack segments and non-crack pseudo-edges. Especially in dense crack network areas, models are prone to errors including boundary overflow, detail adhesion or local fracture. In the GAPs384 dataset, cracks generally present larger widths and clearer morphology with relatively simple background texture, but the dataset still contains challenging fine cracks and background noise in terms of detail and boundary accuracy.

### 3.5. Ablation Experiment and Module Effectiveness Analysis

To further verify the individual contributions of each component in BSA-Net, as well as their synergistic effects in multi-scale fine crack segmentation, a series of ablation experiments were conducted under a unified experimental setting. By progressively incorporating the key components of the proposed model, the influence of different modules on segmentation performance was systematically analyzed, thereby validating the effectiveness and superiority of the overall architectural design.

#### 3.5.1. Quantitative Comparison of Evaluation Metrics

**Figure 8:**
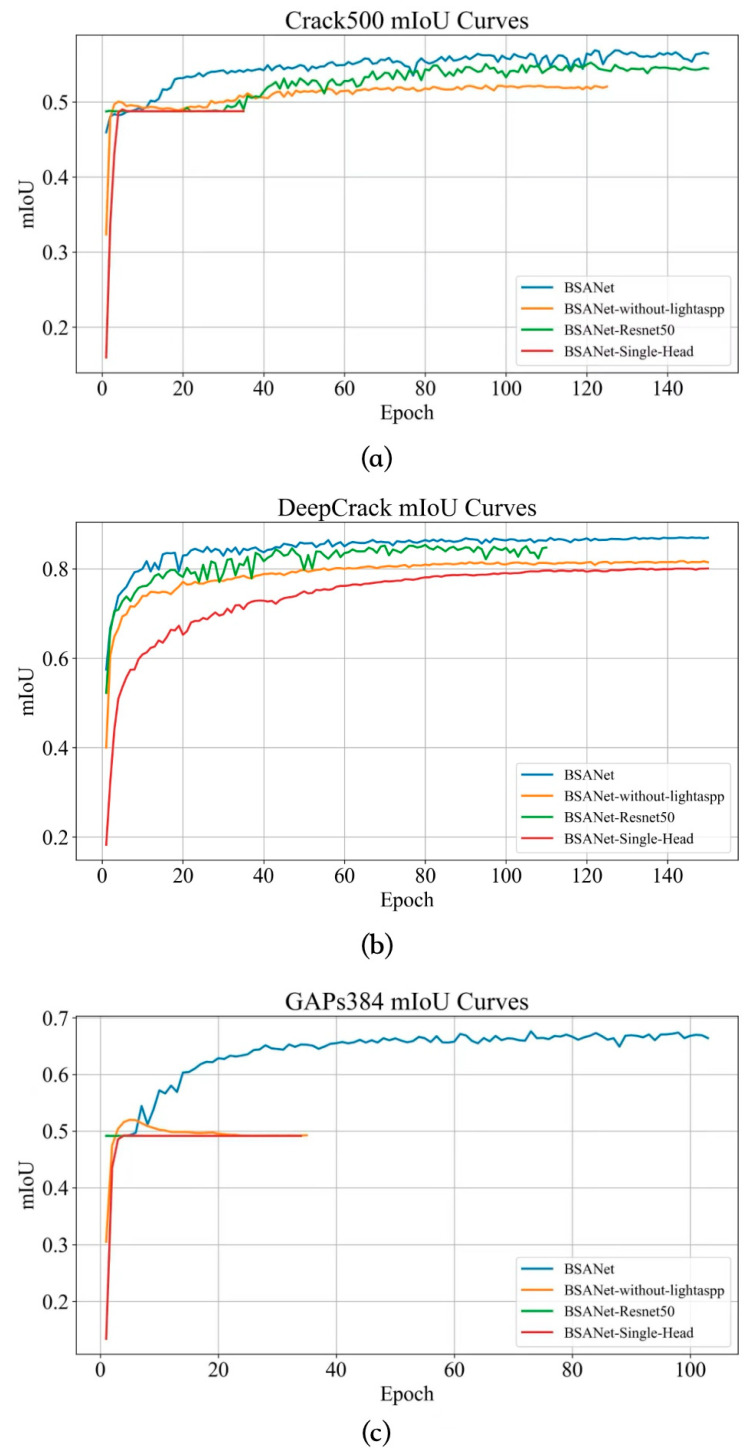
The mIoU curves of evaluation metrics results of the ablation experiment of BSA-Net on three dataset: (**a**) Crack500 dataset; (**b**) DeepCrack dataset; (**c**) GAPs384 dataset.

To ensure a fair comparison, all network variants were trained on the same datasets under identical parameter settings. The resulting mIoU curves with respect to training epochs on the three datasets are shown in Figure 8. Four experimental settings were designed in this study: (1) replacing the backbone of BSA-Net with ResNet50 while keeping all other components unchanged, represented by the green curve; (2) removing the Light-ASPP module from the neck while keeping the remaining components unchanged, represented by the orange curve; (3) replacing the head of BSA-Net with a single-branch detection head while keeping the other components unchanged, represented by the red curve; and (4) the complete BSA-Net model, represented by the blue curve. The quantitative evaluation results of these different module configurations on the three datasets are reported in the corresponding Table 5, Table 6 and Table 7.

Next, the performance of each model of the ablation experiment is analyzed on a dataset-by-dataset basis.

1. Crack500 Dataset

As shown in Table 5, Table 6 and Table 7, the complete BSA-Net achieves the best overall performance on Crack500, with an mIoU of 0.589, an ${\;F}_{1}$ score of 0.343, and an HD95 of 181.37. Compared with the three ablated variants, its superiority is not only reflected in mIoU, but more importantly in its more balanced Precision–Recall trade-off, which indicates a more stable and effective crack detection capability.

When the backbone is replaced with ResNet50, the model performance decreases to an mIoU of 0.565 and an ${\;F}_{1}$ score of 0.273. This suggests that when cracks are fine, the background is complex, and the annotated boundaries are inherently uncertain, a simple convolutional backbone is more likely to learn background-dominated discriminative patterns, leading to insufficient crack representation. Although this variant achieves a slightly higher Precision (0.449) than the complete model (0.388), its Recall (0.196) is much lower than that of the complete model (0.308), indicating a more conservative prediction behavior: false positives are reduced, but at the cost of substantially increased missed detections. For crack inspection, such a bias directly weakens the capability of defect discovery.

After removing Light-ASPP, the model achieves an mIoU of 0.519 and a Precision of 0.501, but its Recall drops sharply to 0.063 and the ${\;F}_{1}$ score decreases to 0.112. This indicates that on datasets such as Crack500, where crack scales vary widely and fine cracks occupy a considerable proportion, the absence of multi-scale context makes it difficult for the network to maintain crack responses in low-contrast regions. Consequently, the model tends to output only a small number of relatively accurate crack fragments, leading to acceptable Precision but a dramatic collapse in Recall. Therefore, the major contribution of Light-ASPP in this setting lies less in improving discriminability alone and more in substantially enhancing detectability and continuity.

The single-branch head almost fails completely, with an mIoU of 0.489, a Precision of 0.047, a Recall of 0.015, an ${\;F}_{1}$ score of 0.023, and an HD95 of 506.23. This result indicates that for elongated targets such as cracks, learning regional consistency and boundary precision through a single pathway easily leads to conflicting optimization objectives. Region segmentation tends to favor merging and smoothing, whereas boundary modeling requires sparse and sharp responses. When both objectives are forced into a single head, they constrain each other, ultimately making it impossible for the model to both localize crack regions stably and preserve boundary structures.

The mIoU convergence curves on Crack500 further confirm these observations. The complete BSA-Net continues to improve throughout training and remains stable at a high performance level in the later epochs, whereas the variants without Light-ASPP and with a single-branch head reach an early plateau. This suggests that structural simplification not only lowers the performance ceiling, but also narrows the feasible optimization space, causing the models to fall into suboptimal solutions more quickly. This is consistent with the nature of crack segmentation: elongated structures require stronger multi-scale semantic support and clearer boundary supervision; otherwise, training is prone to collapse into background-dominant prediction under noise and severe class imbalance.

2. DeepCrack Dataset

On DeepCrack, the complete BSA-Net again achieves the best overall performance, with an mIoU of 0.866, a Precision of 0.924, a Recall of 0.774, an ${\;F}_{1}$ score of 0.842, and an HD95 of 16.07, indicating strong consistency in modeling both crack regions and boundaries. It is noteworthy that the textures in this dataset are generally clearer and the crack morphologies are more typical, so the variants do not exhibit the same extreme collapse observed on Crack500. Nevertheless, the performance differences still show clear structural implications.

When the backbone is replaced by ResNet50, the model obtains an mIoU of 0.853 and an ${\;F}_{1}$ score of 0.837. Although the overall performance decreases slightly, its Recall (0.785) is even marginally higher than that of the complete model (0.774). This suggests that in relatively clear scenarios, ResNet50 can still learn strong crack responses, thereby producing more aggressive recall. However, its Precision is noticeably lower than that of the complete model, indicating that false positives are amplified. In engineering inspection, high Precision is at least as important as Recall, since false positives increase post-processing and manual verification costs. The advantage of BSA-Net on this dataset lies precisely in achieving comparable or even better mIoU and ${\;F}_{1}$ with a higher Precision, demonstrating stronger discriminability and consistency in its learned representation.

After removing Light-ASPP, the model performance decreases to an mIoU of 0.824 and an ${\;F}_{1}$ score of 0.800, with both Precision and Recall declining. This indicates that the contribution of multi-scale context on DeepCrack is reflected in an overall improvement rather than solely in Recall. Since DeepCrack still involves varying crack widths and diverse background textures, multi-scale semantics remain equally important for stabilizing segmentation boundaries and suppressing spurious responses.

The single-branch head also performs worse, with an mIoU of 0.804, an ${\;F}_{1}$ score of 0.773, and an HD95 of 33.94, indicating that even on a relatively easier dataset, boundary-region decoupling still provides a stable structural prior. The boundary branch makes fine crack structures more controllable, while the region branch preserves connectivity and the integrity of the main crack body. Their collaboration helps reduce common errors such as discontinuity, adhesion, and boundary expansion.

The convergence curves on DeepCrack show that all models improve rapidly in the early stage, but the complete BSA-Net remains consistently ahead with smaller fluctuations. This suggests that it not only improves the final performance, but also stabilizes the training process, reflecting the positive role of the proposed architecture in constraining the optimization path.

3. GAPs384 Dataset

On GAPs384, the advantage of the complete BSA-Net is the most pronounced, achieving an mIoU of 0.684, a Precision of 0.592, a Recall of 0.564, an ${\;F}_{1}$ score of 0.577, and an HD95 of 58.48. In contrast, all three structural modifications cause substantial performance degradation, showing clear signs of cross-domain mismatch.

When the backbone is replaced with ResNet50, the model almost fails completely, with an mIoU of 0.494, a Precision of 0.023, a Recall of 0.003, and an ${\;F}_{1}$ score of 0.005. This result suggests that GAPs384 exhibits much stronger domain discrepancies from the other two datasets in terms of imaging conditions, background textures, and crack morphology distributions. A convolutional backbone trained from scratch or with weak priors is therefore insufficient to learn stable crack-discriminative boundaries. In contrast, Hiera-A provides stronger visual priors and adaptive mechanisms, supplying transferable foundational representations for engineering defects such as cracks, which are characterized by limited samples and high noise, thereby substantially improving cross-domain applicability.

After removing Light-ASPP, the model achieves an mIoU of 0.515 and an ${\;F}_{1}$ score of 0.096. Although this is slightly better than the ResNet50 variant, it remains far below the complete model. This indicates that under stronger domain discrepancies, the model requires not only a strong backbone, but also multi-scale semantics to extract cracks from complex backgrounds; otherwise, the predictions become fragmented and are accompanied by severe missed detections.

The single-branch head also performs poorly, with an mIoU of 0.495, an $F_{1}$ score of 0.020, and an HD95 of 536.42, indicating that in more challenging scenarios, the structured constraint introduced by boundary supervision becomes even more indispensable. In engineering images, cracks are often accompanied by interference such as contamination, shadows, and honeycombed surface textures. Without a dedicated boundary branch for fine linear structure modeling, the network can easily submerge cracks into texture noise.

The mIoU curves on GAPs384 further show that the complete BSA-Net continues to improve and stabilizes at a relatively high level, whereas the three variants enter low-level plateaus very early, especially the backbone-replaced and single-head variants, which show almost no effective learning. From an optimization perspective, this further indicates that Hiera-A provides a decisive learnable representation space, while Light-ASPP and BAD introduce task-matched structured inductive biases within that space.

Overall, ablation experiments show that Hiera-A mainly enhances fundamental representation and cross-domain adaptation capabilities, Light-ASPP mainly improves multi-scale contextual response and crack continuity, and BAD primarily resolves the optimization conflict between region extraction and boundary refinement. The three modules do not simply function via superposition; instead, they jointly support the final performance of BSA-Net from three distinct levels respectively: whether the model can learn cracks effectively, whether it can capture complete cracks, and whether it can segment boundaries accurately.

#### 3.5.2. Visual Comparison

**Figure 9:**
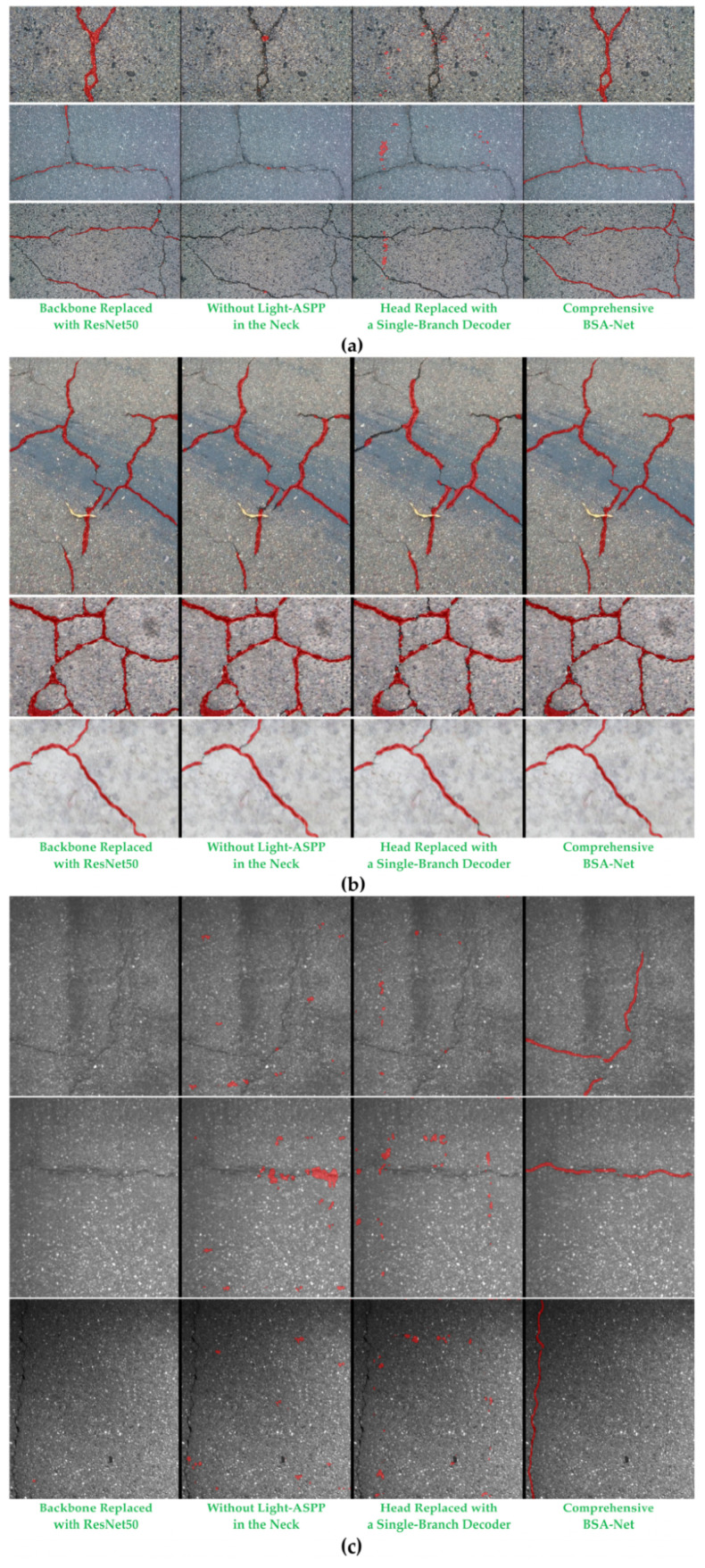
Visual comparison of evaluation metrics results of the ablation experiment of BSA-Net on three datasets of BSA-Net on three dataset: (**a**) Crack500 dataset; (**b**) DeepCrack dataset; (**c**) GAPs384 dataset.

Figure 9 presents the visual comparison results of representative samples from the ablation experiments. From left to right, the results correspond to: (1) BSA-Net with the backbone replaced by ResNet50; (2) BSA-Net without Light-ASPP; (3) BSA-Net with the head replaced by a single-branch decoder; and (4) the complete BSA-Net. The red regions denote the crack foreground predicted by each model.

The three groups of visual examples in Figure 9 show that structural removal or replacement mainly leads to three types of degradation. First, the main crack body becomes discontinuous or breaks at the endpoints, as weak crack segments, bifurcation nodes, and low-contrast regions are more likely to be missed, causing the crack to be segmented into discrete fragments. Second, the predicted crack width becomes unstable and boundary overflow appears, with the predicted regions expanding in texture-disturbed areas around the crack, producing noticeably thicker crack bands and weaker boundary compactness. Third, false crack detections increase, as high-frequency textures, aggregate edges, or local shadow regions in the background are more likely to be mistaken for cracks, leading to scattered noisy points or patch-like false positives.

In contrast, the complete BSA-Net exhibits more consistent suppression of all three degradation patterns. It produces better crack connectivity, more compact boundaries, and fewer erroneous background responses, thereby yielding elongated structural outputs that are more consistent with the true geometric morphology of cracks.

When the backbone is replaced by ResNet50, the model can still respond to the main crack body, but interruptions and missed detections occur more frequently in low-contrast regions and bifurcation areas across all three datasets. For example, in Crack500, the Y-shaped crack structure in sample line 1 is often predicted with unstable connections between the main trunk and the bifurcation, where local holes or discontinuities destroy the topological integrity of the crack. In the transverse crack of sample line 2, weak crack segments are more likely to exhibit fragmented and intermittent responses, making it difficult to preserve a continuous thin-line structure. In DeepCrack, the complex crack intersection in sample line 1 is more prone to local expansion around the intersection node, and the connections of some branches appear less natural. In sample line 2 and line 3, weak crack segments become discontinuous when local contrast changes, producing small missing sections along the crack band. In GAPs384, scattered red patches frequently appear in background-texture regions in sample line 2 and line 3. Even when the true cracks are thin or exhibit weak contrast, the model is more easily triggered by high-frequency textures or local bright spots, resulting in scattered false detections. As a conventional convolutional backbone, ResNet50 focuses more on local texture statistics, and is therefore more susceptible to texture disturbances under complex aggregate backgrounds, as reflected by locally uneven crack widths and slight boundary overflow. By contrast, the complete BSA-Net, equipped with the Hiera-A backbone, demonstrates much stronger structural consistency in the visual results, with weak crack segments being tracked more continuously and bifurcation nodes connected more naturally. This suggests that a conventional convolutional backbone alone is limited in modeling long-range dependencies and preserving weak signals, whereas stronger global representations and task-adaptive mechanisms can more effectively improve crack connectivity and detail stability.

After removing Light-ASPP, the main degradation is reflected in insufficient adaptability to cracks of different widths. On the one hand, thinner or lower-contrast crack regions are more likely to suffer from missed detections and discontinuities; on the other hand, in clearer crack segments, the predicted regions may become locally thicker and the boundary compactness deteriorates. This phenomenon is especially evident in sample line 2 of Crack500, sample line 3 of DeepCrack, and sample line 1 and line 2 of GAPs384. Without multi-scale contextual aggregation, the model becomes less capable of preserving continuous fine crack lines, making gaps more likely to occur in the middle sections of cracks. At the same time, the predicted boundaries become less stable in some clearer crack segments, showing fluctuations in crack bandwidth.

When the head is replaced by a single-branch decoder, the model exhibits a more typical conflict between coverage and compactness. If the model tends to increase crack coverage, boundary overflow and false positives are more likely to occur. Conversely, if it tends to suppress false positives, weak crack segments are more likely to be missed, leading to crack discontinuity. In sample line 1 and line 2 of Crack500, the single-branch predictions are often accompanied by more background noise points or patch-like false detections, while obvious gaps remain in weak crack segments, indicating that a single branch cannot easily satisfy both crack connectivity and boundary compactness. In particular, under the aggregate-texture background of Crack500, particle edges or local high-frequency textures are more likely to be misclassified as cracks, thereby producing scattered false positives. Meanwhile, in low-contrast crack segments, the lack of dedicated constraints for fine boundaries and thin structures leads to discontinuous predictions. In sample line 2 and line 3 of DeepCrack, unnecessary filling trends may appear in narrow background regions between adjacent cracks, or slight discontinuities may occur at crack endpoints, making the crack connectivity inferior to that of the complete model.

The dual-branch decoupling strategy of the complete BSA-Net effectively alleviates these contradictions. The crack branch focuses on structural coverage and connectivity preservation, while the boundary branch emphasizes boundary refinement and false-positive suppression, and the two branches complement each other through feature fusion. As a result, the main crack body remains more continuous, bifurcation nodes appear more natural, and erroneous background responses are significantly reduced, producing outputs that more closely resemble the true crack geometry.

Overall, across the three datasets, the complete BSA-Net consistently demonstrates three advantages: improved connectivity, better boundary compactness, and stronger false-positive suppression. Weak crack segments and endpoints are less prone to breakage, bifurcation nodes preserve more natural topological connections, the predicted crack bandwidth is more stable with less overflow, and scattered background noise or patch-like false positives are substantially reduced. Even in dense aggregate-texture regions, the model is able to maintain relatively clean foreground responses. These advantages arise from the synergy among the backbone, neck, and head: Hiera-A provides a stronger global–local representation foundation, Light-ASPP supplies multi-scale context to improve scale robustness, and BAD decouples regional coverage from boundary refinement during optimization, thereby enabling more stable and trustworthy crack segmentation results.

### 3.6. Ablation Experiment and Effectiveness Analysis of Boundary Branch Loss

To further verify the independent contribution and synergistic effect of the boundary branch loss in the fine multi-scale crack segmentation task, this paper conducts ablation experiments under a unified experimental setup to validate the rationality of the proposed joint loss function design. Two groups of experiments are set up in this study: BSA-Net is trained with an independent region segmentation loss and the proposed joint loss function, respectively. Table 8, Table 9 and Table 10 present the quantitative results of each evaluation metric obtained from different model variants on the test sets of three datasets.

As can be observed, across the three evaluated datasets, after incorporating the boundary branch loss to construct the joint loss function, the mIoU, Precision, Recall, and $F_{1}$ score all achieve improvements of varying degrees, while the HD95 decreases significantly. This demonstrates that after training with the joint loss function formed by introducing the boundary branch loss, the model obtains stronger perception capability for crack boundaries, and is capable of performing more fine-grained crack segmentation tasks.

## 4. Conclusions

This study addressed the challenges of concrete crack segmentation in complex engineering scenarios, where crack targets are typically elongated, exhibit large scale variation, have ambiguous boundaries, and are highly susceptible to background texture interference. To tackle these issues, a Boundary-Sensitive Hybrid Attention Network (BSA-Net) was proposed for fine-grained multi-scale crack segmentation. The proposed framework integrates hierarchical feature encoding, lightweight multi-scale contextual enhancement, and boundary-aware dual-branch decoding, thereby systematically improving traditional crack segmentation networks from three aspects: backbone feature representation, multi-scale semantic enhancement, and collaborative region-boundary optimization during decoding.

In the backbone stage, a hierarchical encoder, Hiera-A, was introduced to overcome the insufficient representation capability of conventional convolutional backbones in complex crack scenes. By progressively encoding local textures, geometric structures, and global contextual relationships at multiple scales, Hiera-A provides a stronger semantic foundation for subsequent feature enhancement and fine-grained decoding. Compared with conventional backbones relying mainly on local convolutional receptive fields, Hiera-A is more effective in modeling the correlation between weak crack regions and the overall crack trend. In addition, a lightweight adaptation mechanism further improves the transferability of the network in crack-related scenarios.

To enhance scale robustness, a Light-ASPP module was designed to perform lightweight multi-scale contextual enhancement on mid- and high-level semantic features. This module improves feature consistency for cracks with significant width variation and scale diversity. Furthermore, a Boundary-Aware Dual-branch decoder (BAD) was introduced to decouple crack region extraction from boundary refinement. Specifically, the segmentation branch focuses on regional integrity and structural connectivity, while the boundary branch emphasizes edge convergence and false-positive suppression. Their fusion enables the network to produce segmentation results that are more consistent with the true geometric contours of cracks.

Extensive experiments conducted on Crack500, DeepCrack, and GAPs384 demonstrate that BSA-Net achieves strong overall performance under different data distributions and scene complexities. On Crack500, BSA-Net achieved mIoU = 0.589, Precision = 0.388, Recall = 0.308, $F_{1}$ score = 0.343, and HD95 = 181.37. On DeepCrack, BSA-Net obtained mIoU = 0.866, Precision = 0.924, Recall = 0.774, $F_{1}$ = 0.842, and HD95 = 16.07. On the more challenging GAPs384 dataset, BSA-Net achieved mIoU = 0.684, Precision = 0.592, Recall = 0.564, $F_{1}$ = 0.577, and HD95 = 58.48. These results indicate that the proposed architecture is not limited to a single dataset or scene type, but exhibits relatively stable and generalizable performance across datasets with different difficulty levels.

The ablation studies further verified the effectiveness and complementarity of the proposed modules. Replacing the backbone with ResNet50 led to consistent performance degradation, particularly on GAPs384, indicating that crack segmentation is highly sensitive to prior feature representation and that Hiera-A provides a stronger global-local representation basis. Removing Light-ASPP caused a substantial drop in recall, especially on Crack500, demonstrating that multi-scale contextual enhancement is crucial for detecting fine and low-contrast cracks. Replacing BAD with a single-branch decoder degraded performance across all datasets, confirming that region extraction and boundary refinement are difficult to optimize simultaneously within a conventional single-branch decoding structure. These findings collectively show that Hiera-A, Light-ASPP, and BAD are all indispensable components of BSA-Net, and that their synergy constitutes the main source of performance improvement.

Visual comparisons further demonstrate that the advantages of BSA-Net are not only reflected in quantitative metrics, but also in the quality of the segmentation outputs. Compared with conventional encoder–decoder architectures, BSA-Net produces segmentation results with better continuity in weak crack regions, more natural preservation of bifurcation structures, stronger boundary compactness, and fewer false positives in complex textured backgrounds. This indicates that the proposed network is capable of learning discriminative patterns that are more consistent with the true geometric characteristics of cracks.

In summary, the proposed BSA-Net, through the collaborative design of Hiera-A, Light-ASPP, and BAD, effectively improves the overall performance of fine-grained multi-scale crack segmentation. The experimental and ablation results confirm both the necessity and complementarity of these components. Therefore, the systematic design strategy adopted in this study, namely from feature encoding to contextual enhancement and boundary optimization, is shown to be both reasonable and effective, and provides a solid foundation for subsequent investigations on model generalization and practical engineering deployment.

## 5. Discussion and Future Work

### 5.1. Discussion

The relatively low foreground Precision and Recall of some baselines should be interpreted in the context of severe foreground-background imbalance. In this study, Precision and Recall are computed for the crack foreground class, whereas mIoU is averaged over foreground and background IoU. When a model predicts most pixels as background, its crack-class Precision and Recall may be extremely low, while the background IoU remains high. Therefore, mIoU alone may not fully reflect foreground segmentation quality in crack segmentation. For this reason, $F_{1}$ score and HD95 are reported together with mIoU.

The proposed method is designed for bridge engineering applications, but practical deployment still requires additional robustness validation. Real bridge inspection images may include strong lighting variation, stains, water marks, dust, surface contamination, motion blur, defocus blur, and annotation uncertainty. Although the extended bridge-crack fusion dataset provides preliminary engineering evidence, larger bridge-specific datasets and controlled degradation tests are needed in future work. Robustness-oriented augmentation, including illumination perturbation, blur simulation, stain-like noise, and texture interference, will also be investigated.

BSA-Net is not positioned as a globally lightweight segmentation network. The overall model is based on a Transformer-derived encoder, so its total parameter count, FLOPs, inference time, and memory consumption may not be lower than those of conventional lightweight CNNs. The lightweight design in this paper refers to module-level efficiency, namely the Light-ASPP context aggregation module and the parameter-efficient Adapter branch. The manuscript therefore avoids broad claims that the entire network is lightweight.

### 5.2. Future Work

Although this study focuses on image-based crack segmentation, structural damage diagnosis is inherently a multi-source perception problem. Vibration- and acoustic-emission-based methods provide complementary information for damage localization and mechanism identification. Future work will therefore explore multimodal structural damage assessment frameworks that integrate UAV-based crack segmentation with vibration response, acoustic emission signals, and other nondestructive testing data. Such multimodal fusion may improve the reliability of bridge condition assessment, especially when surface visual information alone is insufficient to infer internal damage states [35,36,37].

Currently, there are no widely recognized public datasets specifically focused on bridge cracks, and the available datasets used in this study, while including bridge-related data, do not fully capture the unique challenges of bridge crack inspection. To improve the model’s real-world applicability, we plan to build a dedicated dataset with high-precision annotations tailored to bridge inspection scenarios. This specialized dataset will allow for more targeted model training and validation, enhancing the robustness and performance of our BSA-Net model for real-world bridge crack detection tasks. This will be a key direction for the continuation of this research.

Future work will also include more comprehensive comparisons with additional Transformer-based segmentation models such as TransUNet and Swin-UNet, larger bridge-specific datasets, cross-dataset generalization tests, and robustness evaluation under practical engineering disturbances. In addition, model compression, inference speed optimization, and memory-efficient deployment strategies will be investigated to support real-time UAV-based bridge inspection.

## Figures and Tables

**Table 1:** Hardware and Software Configuration.

Experimental Environment	Configuration
CPU	Intel Xeon
GPU	NVIDIA RTX 4090
GPU memory	24 GB
Operating system	Linux Ubuntu 20.04 LTS
CUDA version	CUDA 11.8
Deep-learning framework	PyTorch 2.0.1

**Table 2:** Evaluation metrics results of BSA-Net and the various models on the Crack500 dataset.

Model	mIoU	Precision	Recall	$F_{1}$ Score	HD95
SegFormer	0.556	0.455	0.114	0.183	371.36
U-Net	0.487	0.018	0.002	0.003	595.83
PSPNet	0.529	0.342	0.104	0.159	454.28
DeepLabV3+	0.558	0.390	0.181	0.247	257.24
BiSeNet	0.550	0.385	0.137	0.202	363.54
DANet	0.530	0.318	0.111	0.165	379.94
OCRNet	0.552	0.421	0.179	0.251	307.85
BSA-Net	0.589	0.388	0.308	0.343	181.37

**Table 3:** Evaluation metrics results of BSA-Net and the various models on the DeepCrack dataset.

Model	mIoU	Precision	Recall	$F_{1}$ Score	HD95
SegFormer	0.859	0.876	0.753	0.810	20.89
U-Net	0.856	0.912	0.804	0.854	27.08
PSPNet	0.716	0.738	0.552	0.632	71.20
DeepLabV3+	0.858	0.891	0.801	0.844	16.54
BiSeNet	0.821	0.876	0.729	0.796	22.56
DANet	0.728	0.742	0.585	0.654	57.24
OCRNet	0.816	0.865	0.724	0.788	25.32
BSA-Net	0.866	0.924	0.774	0.842	16.07

**Table 4:** Evaluation metrics results of BSA-Net and the various models on the GAPs384 dataset.

Model	mIoU	Precision	Recall	$F_{1}$ Score	HD95
SegFormer	0.663	0.577	0.458	0.511	68.97
U-Net	0.659	0.642	0.406	0.497	71.53
PSPNet	0.567	0.445	0.180	0.256	165.54
DeepLabV3+	0.673	0.593	0.496	0.540	61.32
BiSeNet	0.489	0.002	0.003	0.003	631.80
DANet	0.572	0.399	0.254	0.311	150.61
OCRNet	0.630	0.486	0.383	0.428	80.06
BSA-Net	0.684	0.592	0.564	0.577	58.48

**Table 5:** Evaluation metrics results of the ablation experiment of BSA-Net on the Crack500 dataset.

Model	mIoU	Precision	Recall	$F_{1}$ Score	HD95
BSA-Net (Backbone Replaced with ResNet50)	0.565	0.449	0.196	0.273	238.36
BSA-Net (Without Light-ASPP in the Neck)	0.519	0.501	0.063	0.112	470.75
BSA-Net (Head Replaced with a Single-Branch Decoder)	0.489	0.047	0.015	0.023	506.23
BSA-Net (Comprehensive)	0.589	0.388	0.308	0.343	181.37

**Table 6:** Evaluation metrics results of **the** ablation experiment of BSA-Net on the DeepCrack dataset.

Model	mIoU	Precision	Recall	$F_{1}$ Score	HD95
BSA-Net (Backbone Replaced with ResNet50)	0.853	0.897	0.785	0.837	18.52
BSA-Net (Without Light-ASPP in the Neck)	0.824	0.828	0.773	0.800	22.06
BSA-Net (Head Replaced with a Single-Branch Decoder)	0.804	0.801	0.746	0.773	33.94
BSA-Net (Comprehensive)	0.866	0.924	0.774	0.842	16.07

**Table 7:** Evaluation metrics results of the ablation experiment of BSA-Net on the GAPs384 dataset.

Model	mIoU	Precision	Recall	$F_{1}$ Score	HD95
BSA-Net (Backbone Replaced with ResNet50)	0.494	0.023	0.003	0.005	622.79
BSA-Net (Without Light-ASPP in the Neck)	0.515	0.121	0.079	0.096	377.24
BSA-Net (Head Replaced with a Single-Branch Decoder)	0.495	0.027	0.012	0.020	536.42
BSA-Net (Comprehensive)	0.684	0.592	0.564	0.577	58.48

**Table 8:** Evaluation Metric Results of BSA-Net Trained with Different Loss Functions on the Crack500 Dataset.

Loss Function	mIoU	Precision	Recall	$F_{1}$ Score	HD95
Single Region Segmentation Loss	0.512	0.363	0.275	0.313	235.62
Joint Loss Function	0.589	0.388	0.308	0.343	181.37

**Table 9:** Evaluation Metric Results of BSA-Net Trained with Different Loss Functions on the DeepCrack Dataset.

Loss Function	mIoU	Precision	Recall	$F_{1}$ Score	HD95
Single Region Segmentation Loss	0.783	0.858	0.697	0.769	35.10
Joint Loss Function	0.866	0.924	0.774	0.842	16.07

**Table 10:** Evaluation Metric Results of BSA-Net Trained with Different Loss Functions on the GAPs384 Dataset.

Loss Function	mIoU	Precision	Recall	$F_{1}$ Score	HD95
Single Region Segmentation Loss	0.601	0.521	0.489	0.504	82.16
Joint Loss Function	0.684	0.592	0.564	0.577	58.48

## Data Availability

The data presented in this study are available in Crack500 at [DOI: 10.1109/TITS.2019.2910595], in DeepCrack at [DOI:10.1109/TIP.2018.2878966], in GAPs384 at [DOI:10.1109/IJCNN.2017.7966101], reference number [27,28,29]. These data were derived from the following resources available in the public domain: [https://github.com/fyangneil/pavement-crack-detection accessed on 30 December 2025], [https://github.com/yhlleo/DeepCrack accessed on 30 December 2025], [https://github.com/RogerQi/GAPS accessed on 30 December 2025].
